# Preparation and Evaluation of Folate Modified PEG-PLLA Nanoparticles Loaded with Lycorine for Glioma Treatment

**DOI:** 10.3390/molecules29051081

**Published:** 2024-02-29

**Authors:** Jieqiong Ding, Jie Su, Binhua Luo, Liqiong Ding

**Affiliations:** 1 Department of Pharmaceutics, School of Pharmacy, Hubei University of Science and Technology, Xianning 437100, China; 2 Department of Physiology, School of Basic Medical Sciences, Hubei University of Science and Technology, Xianning 437100, China

**Keywords:** FA-PEG-PLLA, nanoparticles, lycorine, ROS, apoptosis, NF-κB

## Abstract

Lycorine is a kind of natural active ingredient with a strong antitumor effect. In this study, folate ligand-conjugated polyethylene glycol-block-poly(l-lactide) (PEG-PLLA) nanoparticles (FA-PEG-PLLA NPs) were designed to deliver lycorine to enhance its anti-glioma activity. The successful preparation of the FA-PEG-PLLA polymer was confirmed by ^1^H-NMR, FT-IR, XRD, TGA, and DSC. The optimal formulation for LYC@FA-PEG-PLLA NPs was determined by response surface analysis as follows: sodium dodecyl sulfate (SDS) of 1%, carrier material of 0.03 g, organic phase volume of 3 mL, and ultrasonic power of 20%. The LYC@FA-PEG-PLLA NPs exhibited an encapsulation efficiency of 83.58% and a particle size of 49.71 nm, demonstrating good stability. Hemolysis experiments, MTT assays, and cell scratch assays revealed excellent biocompatibility of FA-PEG-PLLA and superior anti-glioma activity of LYC@FA-PEG-PLLA NPs compared to the raw drug. Additionally, cell apoptosis assays, ROS experiments, and western blot analysis demonstrated that LYC@FA-PEG-PLLA NPs contributed to cell apoptosis by inducing ROS generation and increasing the expression of NF-κB inhibitory protein IκBα. These results suggested that LYC@FA-PEG-PLLA NPs hold promise for glioma treatment.

## 1. Introduction

Glioma is one of the most aggressive and difficult to treat human brain tumors [1]; its 5-year overall survival rate is no more than 35% [2]. At present, the treatment of glioma mainly includes surgical resection, radiotherapy, and chemotherapy [3]. The use of chemotherapy drugs to kill tumor cells is the primary therapeutic method [4].

Lycorine is a natural active alkaloid with various pharmacological effects, including anticancer, antiviral, anti-inflammatory, anti-parasitic, etc., [5,6,7]. Lycorine can significantly inhibit the progression of a variety of tumors, including glioma, and is a potential antitumor drug [8,9,10,11,12,13]. Nevertheless, due to poor solubility and significant side effects, it is difficult for lycorine to achieve sufficient bioavailability and therapeutic effects. Improving the targeting of chemotherapy drugs to tumor tissues to reduce their toxicity and side effects is one of the main goals of current chemotherapy drug research [14]. With the development of nanotechnology, nanoparticles have a wide range of application prospects in the field of drug delivery due to their small particle size, large surface area, and modifiable properties [15]. Nanodrug carriers can be used as a new means for targeted delivery of chemotherapy drugs and reduce their toxic side effects [16,17]. Polyethylene glycol-poly l-lactide acid (PEG-PLLA) nanomaterials are considered to be one of the most effective nanodrug carriers [18].

Furthermore, active targeting has been efficiently exploited to increase nanoparticle internalization by target cells [19]. Here, ligand-anchored drug-loaded NPs can directly interact with receptors present or overly expressed on cancer cell surfaces [20]. Receptor-mediated endocytosis was an endogenous process of cellular uptake of nanomaterials [21,22]. The DNA replication process of malignant tumors requires a large amount of folic acid (FA). As a tumor-related protein, the folate receptor (FR) is overexpressed on the surface of many cancer cells. Therefore, by modifying the folic acid molecule, nanoparticles can be recognized by folate receptors and enter tumor cells through receptor-mediated endocytosis, thus successfully improving the efficacy of chemotherapy drugs [23].

**Figure 1:**
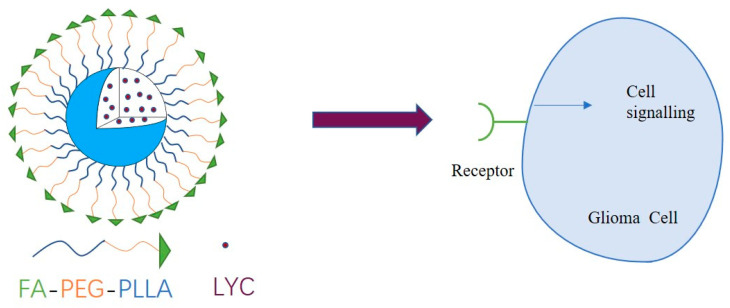
Illustration of the structure and mechanism of action of LYC@FA-PEG-PLLA NPs.

In this paper, folate molecules with specific tumor-targeting properties were linked to PEG-PLLA block copolymers through esterification to form FA-PEG-PLLA drug delivery materials (Figure 1). Subsequently, the carrier material was loaded with lycorine to prepare nanoparticles to achieve a better anti-glioma effect than the parent drug.

## 2. Results and Discussion

### 2.1. Preparation and Characterization of FA-PEG-PLLA

The structure of FA-PEG-PLLA was characterized and confirmed by ^1^H-NMR, FT-IR, XRD, TGA, and DSC. The chemical shift peak of the FA-PEG-PLLA included δ1.58~1.61 (PLLA, -CH_3_), 5.19 (PLLA, -CO-CH(CH_3_)-), 3.66 (PEG, -O-CH_2_-), and δ7~9 (FA, -CH_2_-NH-, Ar-H; Figure S3).

**Figure 2:**
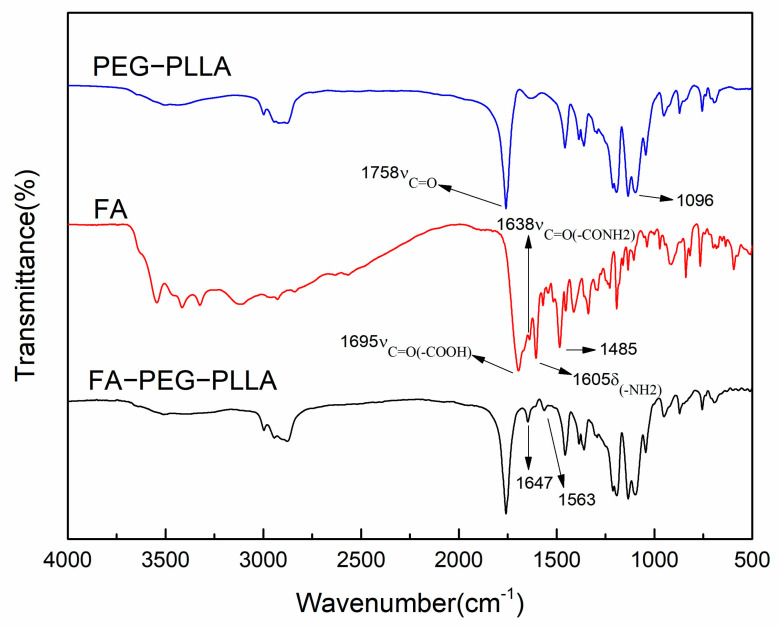
The infrared spectra of FA, PEG-PLLA, and FA-PEG-PLLA.

In the FT-IR spectrum of FA-PEG-PLLA (Figure 2), the 1647 cm^−1^ peak belonged to ν_C=O_ of the amide bond in FA. The peak at 1563 cm^−1^ was the ν_C=C_ of the benzene ring in FA, consistent with the literature [24], confirming that FA had successfully converged with PEG-PLLA.

**Figure 3:**
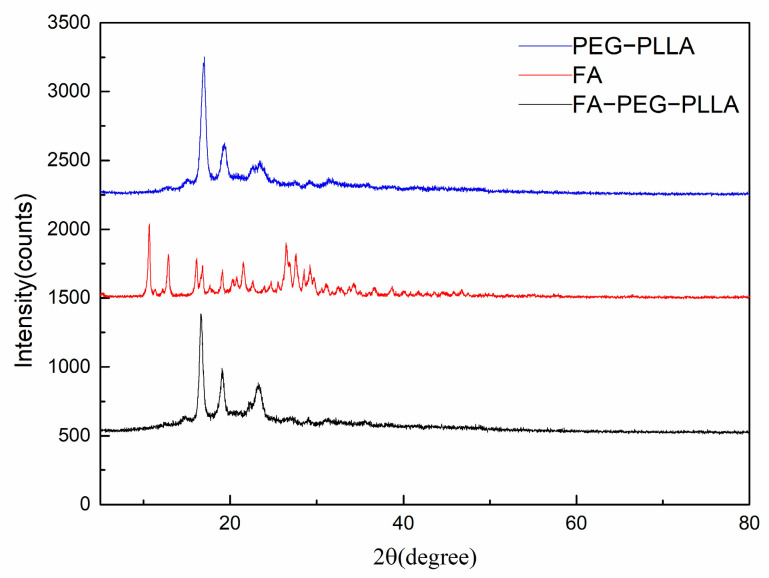
The XRD of FA, PEG-PLLA, and FA-PEG-PLLA.

The crystallinity of FA, PEG-PLLA, and FA-PEG-PLLA were detected using XRD analysis (Figure 3). The principal diffraction peaks of FA centred at 2θ of 10.6, 12.8, 26.4, and 29.30, which confirmed its crystal structure. Compared with PEG-PLLA, the characteristic diffraction peaks of FA were not observed in FA-PEG-PLLA, indicating that FA was in an amorphous state. However, the modification of FA did not significantly influence the diffraction peaks of PEG-PLLA, which indicated that the original crystal structure of PEG-PLLA was unaffected.

**Figure 4:**
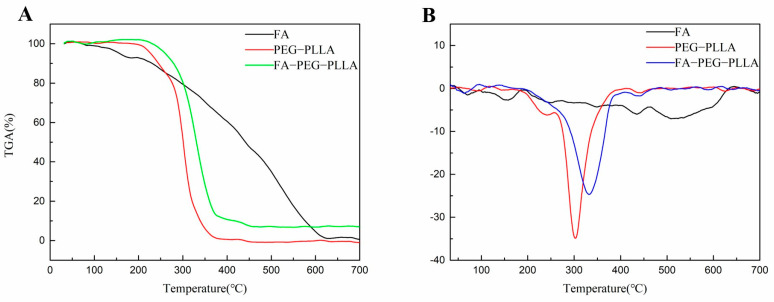
The (**A**) TGA and (**B**) DTG of FA, PEG-PLLA, and FA-PEG-PLLA.

After this, we conducted a thermogravimetric analysis to evaluate the thermal behavior of FA, PEG-PLLA, and FA-PEG-PLLA (Figure 4). PEG-PLLA degradation is a one-step process with an initial degradation temperature of 220 °C and a maximum weight loss temperature of 380 °C, representing complete degradation. In FA-PEG-PLLA, the initial degradation temperature rose to 240 °C, indicating that the addition of FA improves the thermal stability of PEG-PLLA. The weight loss rate of FA-PEG-PLLA reached 89.84% at a temperature of 420 °C.

**Figure 5:**
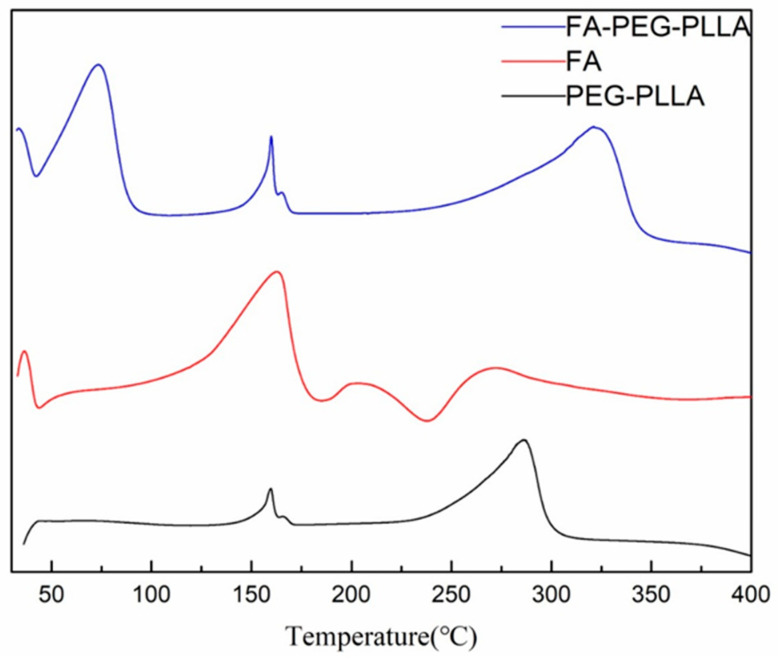
The DSC of FA, PEG-PLLA, and FA-PEG-PLLA.

DSC analysis showed that PEG-PLLA has two endothermic peaks at 160.00 °C and 286.26 °C, while FA-PEG-PLLA has one more endothermic peak at 73.96 °C, indicating that folate was converted to the amorphous form (Figure 5). Overall, the above results proved the covalent binding between FA and PEG-PLLA.

### 2.2. Preparation and Characterization of Nanoparticles

#### 2.2.1. Response Surface Analysis

Design Expert 13.0.1 software was used to perform quadratic polynomial regression fitting for the encapsulation rate and particle size. R^2^ values were 0.7879 and 0.8068, respectively, and the regression equations were as follows:

encapsulation rate (%) = 64.34 − 0.2842A − 6.94B − 0.5225C − 1.86D − 0.5425AB + 0.2050AC + 0.9100AD − 0.3000BC − 1.21BD + 0.2475CD + 12.08A^2^ + 6.02B^2^ + 10.87C^2^ + 5.23D^2^

particle size (nm) = 78.14 − 8.92A + 3.57B + 4.65C + 6.73D + 1.55AB + 0.2600AC − 6.15AD + 5.02BC + 1.20BD + 0.0717CD − 6.02A^2^ − 3.66B^2^ + 1.27C^2^ − 21.94D^2^

**Figure 6:**
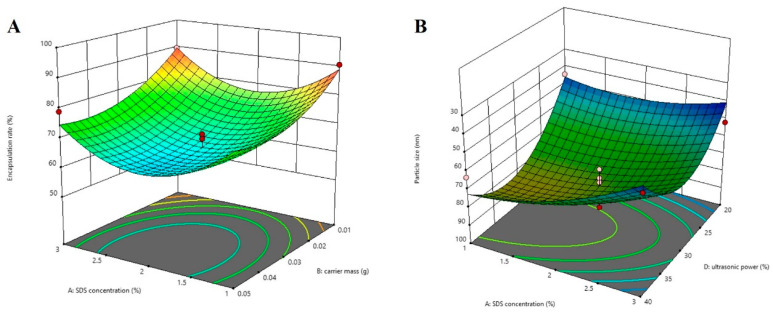
Response surface analysis. (**A**) The influence of SDS concentration and carrier mass on the encapsulation efficiency. (**B**) The influence of SDS concentration and ultrasonic power on the particle size.

The regression coefficient values and variance analysis of each parameter in the quadratic polynomial equation are shown in Table 1. The models of encapsulation rate and particle size fit well, with *p* values of 0.0098 and 0.0057, respectively. Factors B, A^2^, B^2^, and C^2^ had significant effects on the encapsulation rate (*p* < 0.05). Factors A, D, and D^2^ had significant effects on particle size (*p* < 0.05). The results of the variance analysis indicated that carrier mass has a more significant influence on the encapsulation rate. In contrast, sodium dodecyl sulfate (SDS) concentration and ultrasonic power have a greater influence on the particle size. The encapsulation rate gradually increases with the reduction of carrier mass (Figure 6A). At the same time, the particle size decreases with the increase in SDS concentration (Figure 6B), indicating that a high concentration of external emulsifier can reduce interfacial tension, make the nanoparticles more evenly dispersed in the water phase, and form smaller particle size NPs.

#### 2.2.2. Prescription Optimization and Characterization of Nanoparticles

The optimal conditions were predicted by fitting the equation under the condition of an organic phase volume range of 2~4 mL and a particle size range of 39~97 nm, with the minimum concentration of SDS. The optimal prescription of nanoparticles was as follows: (A) 0.0300 g carrier material mass, (B) 1% SDS concentration, (C) 3 mL organic phase volume, and (D) 20% ultrasonic power. The encapsulation rate and particle size of the prepared nanoparticles were detected and are shown in Table 2. The measured values are close to the predicted response values, indicating that the model has a good prediction effect.

**Figure 7:**
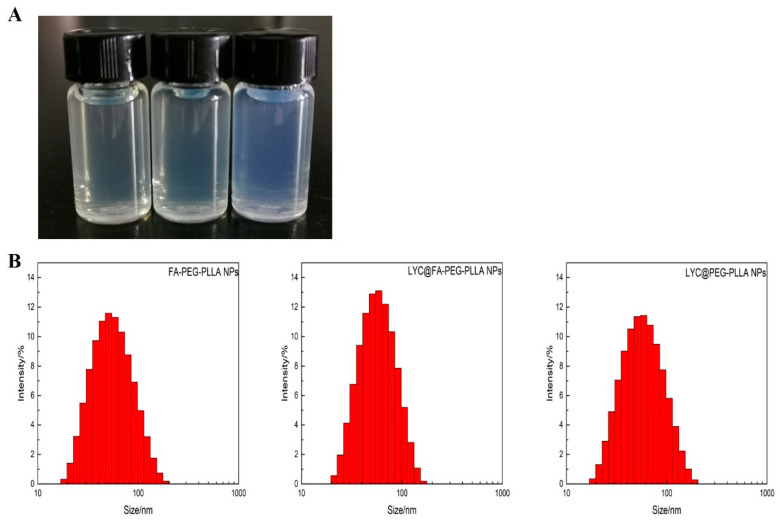
The (**A**) appearance and (**B**) particle sizes of FA-PEG-PLLA NPs, LYC@FA-PEG-PLLA NPs, and LYC@PEG-PLLA NPs.

FA-PEG-PLLA NPs, LYC@FA-PEG-PLLA NPs, and LYC@PEG-PLLA NPs solution presented pale yellow, pale yellow, and blue opalescence, respectively (Figure 7A). The particle sizes were 55.86 ± 5.28 nm, 50.70 ± 1.62 nm, and 56.14 ± 5.18 nm, with PDI of 0.256 ± 0.059, 0.281 ± 0.0627, and 0.273 ± 0.0467, respectively (Figure 7B). These results indicate that the LYC@FA-PEG-PLLA NPs are small in size and narrow in distribution.

**Figure 8:**
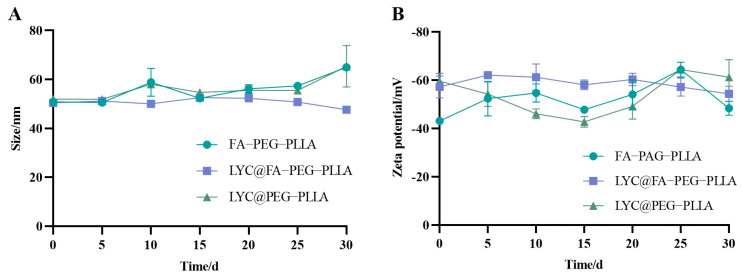
The changes of (**A**) size and (**B**) zeta potential of FA-PEG-PLLA NPs, LYC@FA-PEG-PLLA NPs, and LYC@PEG-PLLA NPs in 30 days.

The stability of the nanoparticles was recorded for 30 days. The results showed that there were no significant changes in the particle size and potential changes (Figure 8), indicating that the nanoparticles could be stably stored within 30 days.

**Figure 9:**
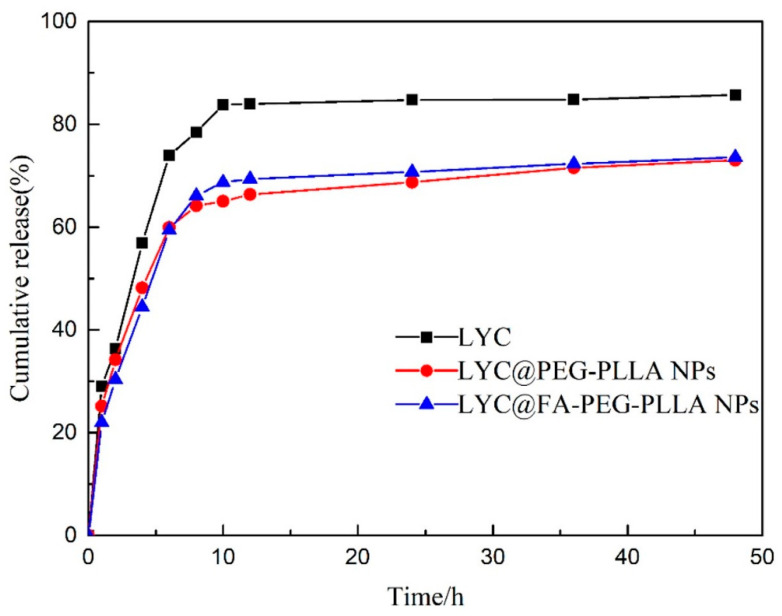
The cumulative release of LYC, LYC@PEG-PLLA NPs, and LYC@FA-PEG-PLLA NPs.

As shown in Figure 9, about 56.90% of LYC was released within 4 h, after which the release rate accelerated. The cumulative release was about 83.79% at 10 h, and then it slowed down. Compared with LYC, the release rates of LYC@PEG-PLLA NPs and LYC@FA-PEG-PLLA NPs were 48.20% and 44.50%, respectively, within 4 h. Further, the cumulative release was 65.05% and 68.71% at 10 h, which was lower than that of LYC. The encapsulation of nanoparticles slowed down the release of the drug, and there was no initial burst release effect. These results indicated that LYC@FA-PEG-PLLA NPs had good release performance under normal physiological conditions.

### 2.3. Hemolytic Assay

**Figure 10:**
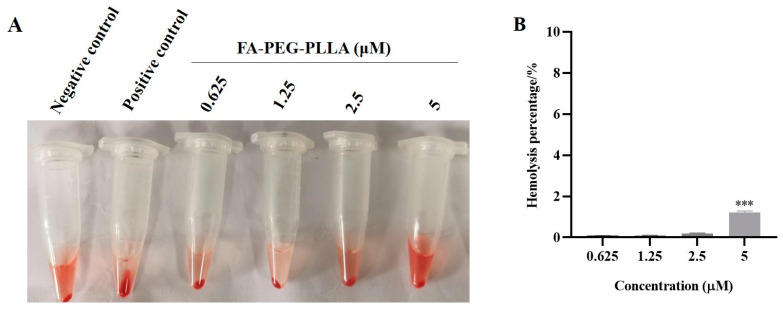
Hemolysis analysis. The (**A**) state and (**B**) hemolysis percentage of red blood cells after treatment with 0.625, 1.25, 2.5, and 5 μM FA-PEG-PLLA NPs. *** *p* < 0.001.

Although nanoparticles have advantages in size and surface properties, intravenous injection may impose the risk of severe red-cell damage, called hemolysis [25]. Therefore, the study of hemolytic activity was one of the most important safety factors for avoiding serious side effects during in vivo drug administration. The biosafety of FA-PEG-PLLA material was investigated by hemolytic assay. After incubation of FA-PEG-PLLA nanoparticles solution with red blood cell suspension for 1 h at different concentrations (0.625, 1.25, 2.5, 5 μM), the absorbance was detected by spectrophotometer; these results are shown in Figure 10. The hemolysis rate of drug-free FA-PEG-PLLA NPs was less than 5%, which indicates a certain level of biosafety.

### 2.4. Cytotoxicity and Cell Migration Assay

**Figure 11:**
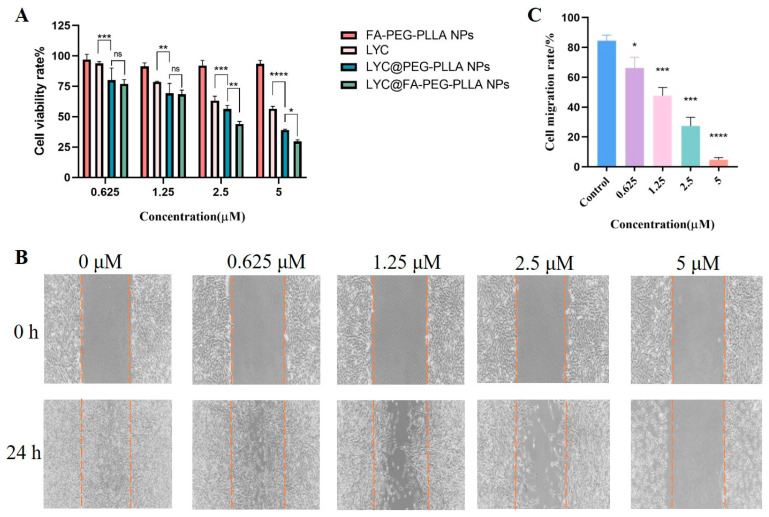
The effect of LYC@FA-PEG-PLLA NPs on cell viability and migration. (**A**) The viability of C6 cells after treatment with 0.625, 1.25, 2.5, and 5 μM FA-PEG-PLLA NPs, LYC, LYC@PEG-PLLA NPs, and LYC@FA-PEG-PLLA NPs for 24 h. (**B**) The migration of C6 cells after treatment with 0.625, 1.25, 2.5, and 5 μM LYC@FA-PEG-PLLA NPs for 24 h. (**C**) The quantitative analysis of (**B**). The blue column represents the control group, the purple column represents 0.625 μM LYC@FA-PEG-PLLA NPs treatment group, the pink column represents 1.25 μM LYC@FA-PEG-PLLA NPs treatment group, the green column represents 2.5 μM LYC@FA-PEG-PLLA NPs treatment group, and the orange column represents 5 μM LYC@FA-PEG-PLLA NPs treatment group. ns means no significance, * *p* < 0.05, ** *p* < 0.01, *** *p* < 0.001, **** *p* < 0.0001.

To determine the effects of nanoparticles on the growth of C6 glioma cells, an MTT assay was used to measure the activity of C6 cells at 0, 0.625, 1.25, 2.5, and 5 μM of nanoparticles and lycorine. FA-PEG-PLLA NPs had little effect on cell survival, whereas LYC@FA-PEG-PLLA NPs (IC_50_: 0.28 μM) were more toxic to cells than LYC@PEG-PLLA NPs (IC_50_: 0.33 μM) and lycorine (IC_50_: 0.56 μM) (Figure 11A). We then conducted the cell scratch assays to further investigate the effects of LYC@FA-PEG-PLLA NPs on the migration of C6 glioma cells. As shown in Figure 11B,C, the migration rate of cell treatment with LYC@FA-PEG-PLLA NPs at different concentrations (0.625, 1.25, 2.5, and 5 μM) was 66.09%, 47.60%, 27.37%, and 4.82%, respectively. This was significantly lower than the control group (84.59%). These results indicate that LYC@FA-PEG-PLLA NPs can significantly inhibit C6 cell migration at concentrations of 0.625–5 μM, which is consistent with the trend of MTT assay.

### 2.5. Cell Apoptosis Assays

**Figure 12:**
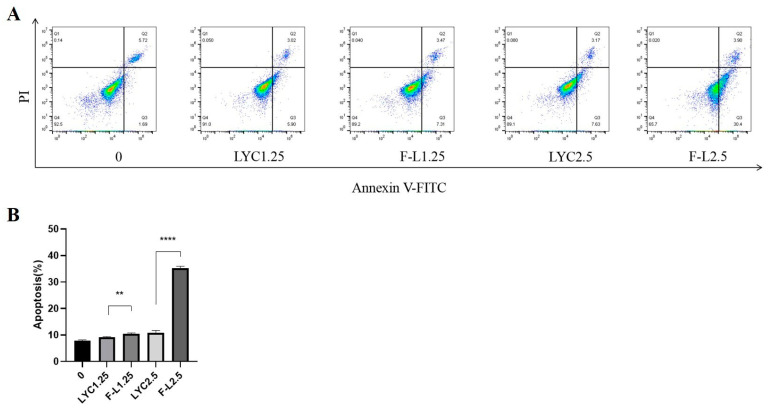
The effect of LYC@FA-PEG-PLLA NPs on cell apoptosis. (**A**) Flow cytometry histograms of C6 cells treated with 1.25 and 2.5 μM of LYC and LYC@FA-PEG-PLLA NPs (F-L) for 24 h. (**B**) The quantitative analysis of (**A**). ** *p* < 0.01, **** *p* < 0.0001.

Apoptosis is a key mechanism of drug-induced anticancer effects. Flow cytometry was used to determine the apoptosis of LYC@FA-PEG-PLLA NPs and LYC-treated cells, as shown in Figure 12. After 24 h, compared with 7.80% in the control group, the apoptosis rates of cells treated with 1.25 μM LYC and LYC@FA-PEG-PLLA NPs were 9.15% and 10.47%, respectively. Under 2.5 μM LYC and LYC@FA-PEG-PLLA NPs, the apoptosis rates of C6 cells were 10.92% and 35.20%, respectively, indicating that the apoptosis effect induced by LYC@FA-PEG-PLLA NPs was higher than that induced by lycorine. Notably, the apoptosis rate was significantly higher with 2.5 μM LYC@FA-PEG-PLLA NPs than with 2.5 μM lycorine, as well as with 1.25 μM NP, suggesting that LYC@FA-PEG-PLLA NPs concentration-dependently induced apoptosis and confirmed that LYC@FA-PEG-PLLA NPs could promote lycorine accumulation in cells and kill more cancer cells.

### 2.6. Reactive Oxygen Species (ROS) Experiments and Western Blot Analysis

**Figure 13:**
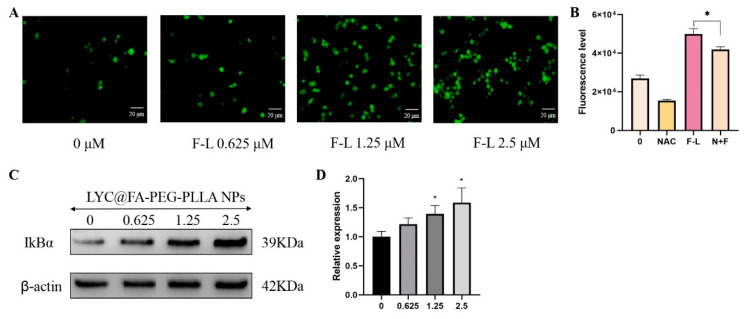
The anti-glioma mechanism of LYC@FA-PEG-PLLA NPs. (**A**) The fluorescence image and (**B**) the fluorescence intensity of C6 cells stained with DCFH-DA after treatment with 0.625, 1.25, and 2.5 μM LYC@FA-PEG-PLLA NPs (F-L) for 2 h. (**C**) The expression of IκBα of C6 cells after treatment with 0.625, 1.25, and 2.5 μM LYC@FA-PEG-PLLA NPs (F-L) for 24 h. (**D**) The quantitative analysis of (**C**). * *p* < 0.05.

Oxidative damage mediated by ROS is the main mechanism of apoptosis induced by anticancer drugs [26]. Fluorescence microscopy and flow cytometry were used to detect fluorescence intensity after LYC@FA-PEG-PLLA NPs treatment to study ROS levels in C6 cells. As shown in Figure 13A, with the increase in LYC@FA-PEG-PLLA NPs concentration, intracellular fluorescence intensity of reactive oxygen species gradually increased. Compared with the untreated group, the level of intracellular reactive oxygen species was significantly increased under 2.5 μM LYC@FA-PEG-PLLA NPs treatment, while the level of intracellular ROS was significantly decreased when NAC (a ROS inhibitor) was added (Figure 13B). It was confirmed that LYC@FA-PEG-PLLA NPs could increase the level of intracellular reactive oxygen species. Following this, we detected the expression of NF-κB pathway-associated protein IκB in LYC@FA-PEG-PLLA NPs treated C6 glioma cells. It was found that LYC@FA-PEG-PLLA NPs treatment increased the expression of the inhibitory protein of NF-κB, IκB (Figure 13C,D). In conclusion, LYC@FA-PEG-PLLA NPs can induce apoptosis of C6 cells by increasing the production of ROS and the expression of IκB protein, thus producing anti-glioma effects.

## 3. Materials and Methods

### 3.1. Materials

L-Lactide (LLA) was purchased from Daigang Biotechnology Co., Ltd. (Jinan, China) and was recrystallized from dry ethyl acetate before use. Polyethylene glycol (PEG, Mw = 6000) and dimethyl sulfoxide (DMSO) were purchased from Sinopharm Chemical Reagent Co., Ltd. (Shanghai, China). Lycorine hydrochloride (LYC), Folic acid (FA), 1-(3-dimethylaminopropyl)-3-ethylcarbodiimide (EDCI), and stannous octoate [Sn(Oct)_2_] were purchased from Aladdin Biochemical Technology Co., Ltd. (Shanghai, China). The 4-dimethylamino pyridine (DMAP) was purchased from Bide Pharmatech Ltd. (Shanghai, China). Pyridine was purchased from Tianli Chemical Reagent Co., Ltd. (Tianjin, China). N-acetyl cysteine (NAC) was obtained from Sigma, MO, USA.

The 3-(4,5)-dimethylthiahiazo (-z-y1)-3,5-di-phenytetrazoliumromide (MTT) was obtained from Solarbio, Beijing, China. Dichlorodihydrofluorescein diacetate (DCFH-DA) fluorescent probe was obtained from the Beyotime Institute of Biotechnology, Haimen, China. Annexin V-FITC/PI (Propidium Iodide) apoptosis detection kit was purchased from Yeasen Biotechnology, Shanghai, China. Antibodies against β-actin and IkBα were purchased from ABclonal Technology Inc., Wuhan, China.

### 3.2. Synthesis and Characterization of FA-PEG-PLLA

#### 3.2.1. Synthesis of PEG-PLLA

The ring-opening polymerization method was used to synthesize PEG-PLLA. Under the condition of nitrogen, 4.7514 g recrystallized L-LA, 1.9797 g PEG6000, and 0.1337 g Sn(Oct)_2_ (molar ratio of the three substances is 100:1:1) were heated at 120 °C for 2 h in a 50 mL round-bottom flask equipped with a magnetic stirrer. When the crude product cooled to room temperature, a small amount of chloroform was used to dissolve it. After this, the desired product was gradually precipitated and washed with a small amount of anhydrous ether twice. Finally, the resulting product was 6.3533 g, and the yield was 95.81% after being dried at 40 °C to a constant weight. The reaction equation is shown in Figure S1 in the Supplementary Materials.

#### 3.2.2. Synthesis of FA-PEG-PLLA

A 10 mg measure of folic acid (FA) powder was dissolved with 5 mL DMSO in a beaker, and then 4.3 mg EDCI and 2.8 mg DMAP (molar ratio of the FA: EDCI: DMAP = 1:1:1) were added to the solution. Afterward, for activation of folate, the mixture solution was stirred at room temperature for 24 h, which was followed by the addition of 45.4 mg PEG-PLLA (molar ratio of the FA: PEG-PLLA = 1:1) and 20 μL pyridine to the mixture solution, and the reaction was continued overnight under the dark condition. The reaction mixture was purified by a dialysis bag (M_W_:14,000) with ultra-pure water as the dialysis medium. DMSO and byproducts were removed using dialysis for 24 h. The product FA-PEG-PLLA was then obtained as a slight yellow powder through lyophilization from the residual solution (yield 39.23 mg, 84.62%). The reaction equation is shown in Figure S2 in the Supplementary Materials.

#### 3.2.3. Characterization of FA-PEG-PLLA

**^1^H-NMR**. To evaluate the successful synthesis of FA-PEG-PLLA, a Bruker Avance-400 nuclear magnetic spectrometer was used for the determination of FA-PEG-PLLA using ^1^H-NMR. The samples were dissolved with CDCl_3_, and tetramethylsilane was used as the internal standard for determination at a resonance frequency of 400 MH_Z_.

**FT-IR**. Spectroscopic evaluations of the samples were analyzed using a PerkinElmer FTIR spectrophotometer. FA, PEG-PLLA, and FA-PEG-PLLA were processed using the potassium bromide (KBr) compression method, and the scanning analysis was performed in the wave number range of 4000 cm^−1^–500 cm^−1^.

**XRD**. The crystallization properties of FA, PEG-PLLA, and FA-PEG-PLLA were scanned using an X-ray diffractometer (XRD-6100). The relevant operation steps and testing conditions are as follows: the sample was poured into the sample pool, flattened, and put into the sample seat, and then the machine door was closed for testing. Tube voltage: 40 KV; Tube current: 30 mA; Scanning range: 5~100 °C; Scanning speed: 10 °C/min; Sweep step: 0.1 °C.

**TGA**. The thermal stability of FA, PEG-PLLA, and FA-PEG-PLLA polymers was analyzed using a thermogravimetric analyzer (TG 209 F3, Netzsch, Germany). The related determination conditions and operation are as follows: the zero adjustment was carried out with a blank Al_2_O_3_ crucible, and the sample was put into the crucible. The temperature was raised to 20 °C/min in a nitrogen atmosphere, and the end temperature was 700 °C.

**DSC**. Scanning analysis of FA, PEG-PLLA, and FA-PEG-PLLA was performed using a DSC-200-F3 differential scanning calorimeter. An empty aluminum crucible was used as a reference. The weighed FA, PEG PLLA, and FA-PEG-PLLA (4~9 mg) were placed in another aluminum crucible under nitrogen. The scanning range is 30~400 °C, and the heating rate is 10 °C/min.

### 3.3. Preparation and Characterization of Nanoparticles

#### 3.3.1. Preparation of Nanoparticles

Folate ligand-conjugated polyethylene glycol-block-poly(l-lactide) (PEG-PLLA) loaded with lycorine nanoparticles (LYC@FA-PEG-PLLA NPs) were prepared using an emulsion-solvent evaporation method. The optimal formulation process was determined using the Box–Behnken design, which examines correlations between variables and responses and the selection of the optimum formulation [27,28]. According to the previous results of the single-factor experiment, external emulsifier SDS concentration (A), carrier mass (B), organic phase volume (C), and ultrasonic power (D) were selected as the factors to investigate, and encapsulation rate and particle size were selected as the response values. The Design Expert 13.0.1 software was used to design response surface tests with four factors and three levels. The factor levels are shown in Table 3. A total of 29 tests were conducted to analyze the response surface test results, as shown in Table 4.

#### 3.3.2. Characterization of Nanoparticles

##### Particle Size, Zeta Potential, Encapsulation Rate, and Drug Loading

A volume of 50 μL Lyc@FA-PEG-PLLA NPs was diluted with 3 mL ultra-pure water, then the particle size and potential of the nanoparticles were evaluated using a laser particle size analyzer. The encapsulation rate and drug loading of nanoparticles were determined using the UV method at a wavelength of 390 nm (characteristic absorption band of lycorine). A volume of 500 μL Lyc@FA-PEG-PLLA NPs solution was obtained with a pipette and centrifugated twice at 10,000 rpm for 15 min each time in the ultrafiltration tube (molecular weight 3 kDa). The filtrate outside the ultrafiltration tube was collected and diluted to 10 mL with methanol to determine its absorbance. The encapsulation rate and drug loading of Lyc@FA-PEG-PLLA NPs are obtained according to Equations (1) and (2) as follows:

(1) $$Encapsulation\;efficiency,EE\%=\frac{m_{drug\;of\;the\;nanoparticles}}{m_{total\;of\;drug}}$$

(2) $$Loading\;efficiency,LE\%=\frac{m_{drug\;of\;the\;nanoparticles}}{m_{polymer}+m_{drug}}$$

##### In Vitro Lycorine Release Profiles

Measures of 2 mL lycorine, LYC@FA-PEG-PLLA, and LYC@PEG-PLLA nanoparticles solution were placed into a dialysis bag, respectively. After this, the dialysis bag was submerged fully into 50 mL PBS (pH = 7.4) at 37 °C, and the shaking speed was kept at 100 rpm under dark conditions. At different time intervals, 3 mL of samples were taken out and replaced with an equal volume of PBS. The release of lycorine was determined using the UV method at a wavelength of 390 nm.

### 3.4. Hemolytic Assay

To assess the potential of FA-PEG-PLLA NPs for application in vivo (e.g., intravenous injection), hemocompatibility was evaluated through a hemolysis approach. A 2 mL volume of fresh blood was taken from the abdominal aorta of Sprague–Dawley (SD) rats and placed into the vacuum tubes. After this, the blood was centrifuged at 1000 rpm at 4 °C for 10 min, and the red blood cell precipitation was obtained. The red blood cells were washed with normal saline several times until the upper layer was clear, then the concentration of red blood cell suspension was adjusted to 2% (*v*/*v*) with normal saline. A 150 μL volume of 2% red blood cell suspension was added into the 150 μL different concentrations of FA-PEG-PLLA (0.625, 1.25, 2.5, 5 μM) and incubated at 37 °C for 1 h. The cells were then centrifuged at 1000 rpm at 4 °C for 10 min, and the absorbance of the supernatant was measured at 540 nm using a spectrophotometer. Normal saline and ultrapure water were used as the negative and positive controls. The hemolysis percentage was then calculated using the following Equation:

$$Hemolysis\;percentage=\frac{{OD}_{sample}-{OD}_{negative}}{{OD}_{positive}-{OD}_{negative}}\times 100\%$$

### 3.5. Cell Culture

C6 cells were obtained from ATCC. The cells were cultured in a DMEM medium supplemented with 10% fetal bovine serum and a 1% penicillin and streptomycin solution in a cell incubator containing 5% CO_2_ at 37 °C.

### 3.6. MTT Assay

A MTT assay was used to evaluate the effect of LYC@FA-PEG-PLLA NPs, LYC@PEG-PLLA NPs, LYC, and FA-PEG-PLLA NPs on cell viability. Briefly, C6 cells were seeded into 96-well plates at a density of 1 × 10^5^ per well and incubated for 24 h. After medium removal, different concentrations of LYC@FA-PEG-PLLA NPs, LYC@PEG-PLLA NPs, and LYC and FA-PEG-PLLA NPs solutions were added, and the cells were further incubated at 37 °C for 24 h. After this, a 20 μL MTT solution (5 mg/mL) was added and incubated for 4 h followed by the addition of DMSO to dissolve the formazan precipitate. Finally, the absorbance at 490 nm was recorded using a Spark & Infinite 200 pro plate reader (TECAN, Männedorf, Switzerland).

### 3.7. Cell Scratch Assays

The effects of LYC@FA-PEG-PLLA NPs on the migration of C6 cells were investigated using cell scratch assays. C6 cells were inoculated into the 6-well plate with 2 × 10^5^ cells per well. When the cell density reached about 80%, a straight scratch was produced by a sterile pipette on the cell monolayer, and the cells were gently washed with 1 mL PBS. After this, multiple concentrations (0.3125, 0.625, 1.25, 2.5 μM) of LYC@FA-PEG-PLLA NPs were added to the cells. Finally, the migration distance of C6 cells was measured at 0 and 24 h, respectively.

### 3.8. Cell Apoptosis Assays

The apoptosis of C6 cells was detected by Annexin V-FITC/PI staining. C6 cells were inoculated into 12-well plates (1 × 10^5^ cells/well) and cultured for 24 h. After treatment with different concentrations of LYC and LYC@FA-PEG-PLLA NPs (1.25, 2.5 μM) for 24 h, the cells were collected and washed with PBS and stained with Annexin V-FITC/PI kit (Yeasen Biotechnology, Shanghai, China) according to the manufacturer’s instructions. The working concentrations of Annexin V-FITC and PI were 5% and 10%, respectively. Finally, the samples were analyzed using the Accuri™ C6 Plus flow cytometer (BD Biosciences, San Jose, CA, USA), and FlowJo V10 software was used for data analysis.

### 3.9. Reactive Oxygen Species (ROS) Experiments

The ROS Assay kit (Beyotime Institute of Biotechnology, Shanghai, China) was used to detect the level of reactive oxygen species in C6 cells. (1) ROS dyeing: C6 cells were seeded into a 12-well plate at a density of 2 × 10^5^ cells per well. After incubation for 24 h, the cells were treated with 0, 0.625, 1.25, and 2.5 μM LYC@FA-PEG-PLLA NPs for 2 h. The cell culture medium was then abandoned, and the cells were gently washed with PBS. Next, the cells were incubated in darkness for 30 min with 200 μL DCFH-DA reagent (10 μM) and then washed with PBS once. The ROS levels were recorded under a fluorescence microscope (Olympus Corporation, Tokyo, Japan). (2) Flow cytometry for ROS: C6 cells were seeded into a 12-well plate at a density of 2 × 10^5^ cells per well. After 24 h, the cells were treated with 2.5 μM LYC@FA-PEG-PLLA NPs and 10 mM NAC (ROS inhibitor) for 2 h, and the cell culture medium was abandoned. Next, 200 μL pancreatic enzyme digestion solution was added, and the cells were collected by centrifugation at 1000 rpm for 3 min. Subsequently, the cells were incubated in darkness for 30 min with 200 μL DCFH-DA (10 μM) reagent. After this, the cells were washed twice with PBS. Finally, the fluorescence intensity was measured using the Accuri™ C6 Plus flow cytometer (BD Biosciences, Franklin Lakes, NJ, USA).

### 3.10. Western Blot Analysis

Western blot was performed as described [29]. Briefly, after treatment with LYC@FA-PEG-PLLA NPs (0, 0.625, 1.25, 2.5 μM) for 24 h, cells were collected and lysed with RIPA lysis buffer (Radio immunoprecipitation assay buffer). The samples were quantified, and 5–10% SDS-PAGE gels were used to separate the protein. After this, the separated protein was transferred onto polyvinylidene fluoride (PVDF) membranes (Millipore, Burlington, MA, USA), and 5% skimmed milk was used to seal the membranes at room temperature for 1 h. After they were washed with PBS, the membranes were incubated overnight with the specific antibodies at 4 °C, followed by incubation for 1 h with the secondary antibody at room temperature. Finally, the results were observed through the Chemiluminescence image system (Bio-Rad, Hercules, CA, USA).

### 3.11. Statistical Analysis

Statistical analysis was conducted using a *t*-test. The statistical difference was expressed as * *p* < 0.05, ** *p* < 0.01, *** *p* < 0.001, **** *p* < 0.0001.

## 4. Conclusions

In this study, we successfully synthesized FA-PEG-PLLA polymer and obtained the optimal formulation of LYC@FA-PEG-PLLA NPs using response surface design. The prepared LYC@FA-PEG-PLLA NPs had good stability and biocompatibility and significantly improved the ability of lycorine to inhibit the proliferation, migration, and induction of apoptosis in glioma. Our results indicated that the LYC@FA-PEG-PLLA NPs have the potential to be the carrier for the targeted delivery of lycorine to glioma cells.

## 5. Future Perspective

Folate-conjugated PEG-PLLA nanoparticles loaded with lycorine significantly improve the anti-glioma activity of lycorine with low toxicity and high safety. Therefore, LYC@FA-PEG-PLLA NPs have great potential for glioma treatment and clinical translation. However, our conclusions rely only on in vitro studies, and further in vivo experiments are needed to demonstrate the anti-glioma effects of LYC@FA-PEG-PLLA NPs and validate their possible clinical applications. In addition, the purity of FA-PEG-PLLA must be further determined using high-performance liquid chromatography and mass spectrometry, and the characterization results of LYC@FA-PEG-PLLA NPs need to be further improved, including microscopic images of nanoparticles.

## Figures and Tables

**Table 1:** Regression coefficient and variance analysis for each parameter of the model.

Source	Encapsulation Rate			Particle Size		
	F-Value	*p*-Value		F-Value	*p*-Value	
Model	3.72	0.0098	significant	4.18	0.0057	significant
A: SDS concentration	0.0237	0.8799		10.06	0.0068	
B: carrier mass	14.13	0.0021		1.61	0.2248	
C: organic phase volume	0.0801	0.7813		2.74	0.1202	
D: ultrasonic power	1.02	0.3302		5.73	0.0313	
AB	0.0288	0.8677		0.1015	0.7548	
AC	0.0041	0.9498		0.0028	0.9582	
AD	0.0810	0.7801		1.59	0.2273	
BC	0.0088	0.9266		1.06	0.3205	
BD	0.1432	0.7108		0.0603	0.8096	
CD	0.0060	0.9394		0.0002	0.9885	
A^2^	23.15	0.0003		2.47	0.1381	
B^2^	5.75	0.0310		0.9165	0.3546	
C^2^	18.75	0.0007		0.1105	0.7446	
D^2^	4.34	0.0560		32.89	<0.0001	
Lack of Fit	3.57	0.1158	not significant	1.50	0.3717	not significant

**Table 2:** The predicted and measured responses of the optimal formulation of nanoparticles.

Evaluation Index	Predicted Value	Measured Value	Deviation Rate%
Encapsulation rate/%	84.77	83.58	−1.40
Particle size/nm	46.23	49.71	7.52

**Table 3:** Box–Behnken factor level table.

Factors	Level		
	−1	0	1
A: SDS concentration	1%	2%	3%
B: carrier mass	0.01 g	0.05 g	0.1 g
C: organic phase volume	2 mL	3 mL	4 mL
D: ultrasonic power	20%	30%	40%

**Table 4:** Box–Behnken test design and results.

Std	Run	Factor 1 A: SDS Concentration%	Factor 2 B: Carrier Mass/g	Factor 3 C: Organic Phase Volume/mL	Factor 4 D: Ultrasonic Power%	Response 1: Encapsulation Rate%	Response 2: Particle Size/nm
13	1	2	0.01	2	30	90.52	73.34
1	2	1	0.01	3	30	90.57	71.12
27	3	2	0.03	3	30	67.25	73.67
14	4	2	0.05	2	30	81.29	66.39
18	5	3	0.03	2	30	80.93	59.84
20	6	3	0.03	4	30	83.20	70.24
16	7	2	0.05	4	30	76.75	90.48
10	8	3	0.03	3	20	83.91	51.90
19	9	1	0.03	4	30	82.11	97.24
4	10	3	0.05	3	30	78.94	55.80
3	11	1	0.05	3	30	82.21	73.60
2	12	3	0.01	3	30	89.47	47.12
8	13	2	0.03	4	40	81.20	62.77
17	14	1	0.03	2	30	80.66	87.88
6	15	2	0.03	4	20	84.35	43.07
21	16	2	0.01	3	20	83.20	45.38
5	17	2	0.03	2	20	85.90	39.22
28	18	2	0.03	3	30	59.55	68.72
24	19	2	0.05	3	40	54.43	72.91
12	20	3	0.03	3	40	84.60	45.65
11	21	1	0.03	3	40	82.98	63.06
25	22	2	0.03	3	30	64.12	76.18
29	23	2	0.03	3	30	61.91	81.46
7	24	2	0.03	2	40	81.76	58.64
26	25	2	0.03	3	30	68.85	90.66
23	26	2	0.01	3	40	79.22	57.76
9	27	1	0.03	3	20	85.93	44.71
22	28	2	0.05	3	20	63.25	55.75
15	29	2	0.01	4	30	87.18	77.36

## Data Availability

Data are contained within the article and Supplementary Materials.

## Supplementary Materials

The following supporting information can be downloaded at: https://www.mdpi.com/article/10.3390/molecules29051081/s1, Figure S1: The synthesis route of PEG-PLLA; Figure S2: The synthesis route of FA-PEG-PLLA; Figure S3: The ^1^ H-NMR spectrum of FA-PEG-PLLA.
