# Supplementary material for: Preparation and Evaluation of Folate Modified PEG-PLLA Nanoparticles Loaded with Lycorine for Glioma Treatment
Source: Molecules. 2024 Feb 29;29(5):1081. doi: 10.3390/molecules29051081 (PMC10934019; doi:10.3390/molecules29051081)
Supplement: Supplementary file 1 [file molecules-29-01081-s001.zip › molecules-2856050-supplementary.pdf]

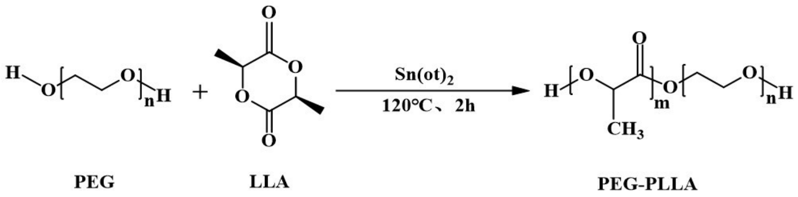

Figure S1. The synthesis route of PEG-PLLA.

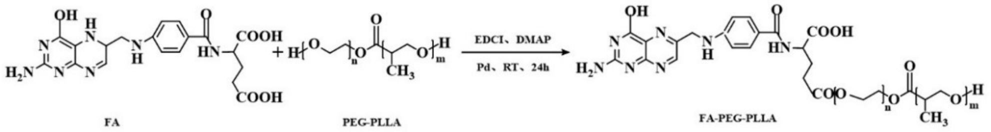

Figure S2. The synthesis route of FA-PEG-PLLA.

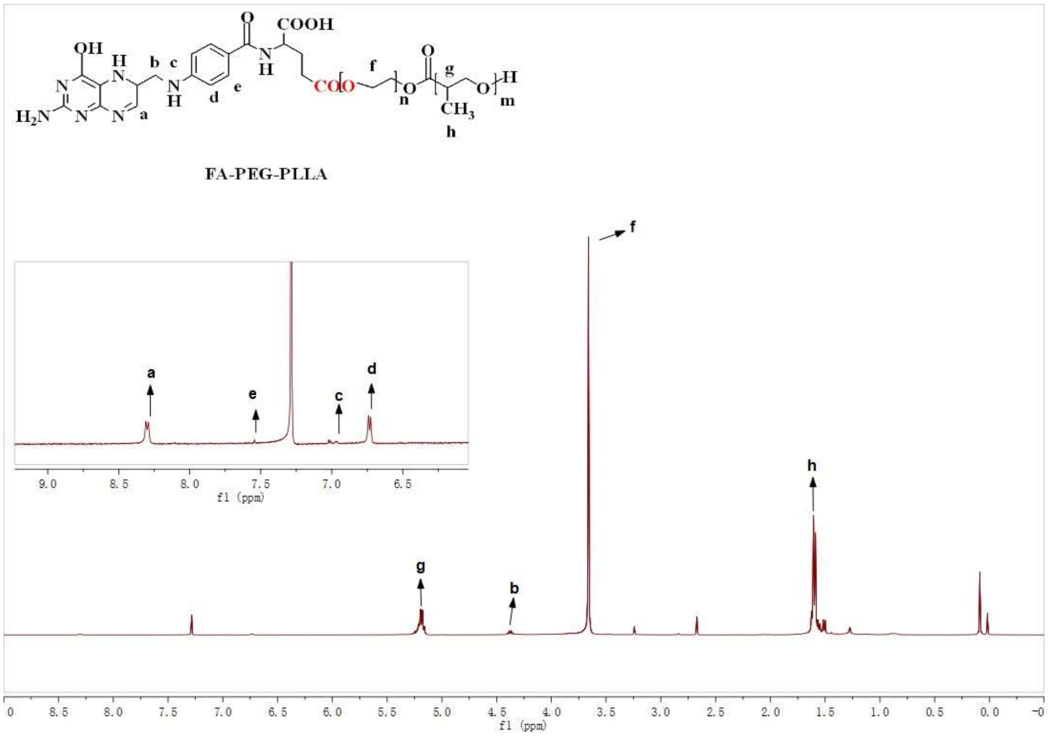

Figure S3. The ^{1}H-NMR spectrum of FA-PEG-PLLA.
